# Nurses’ Experiences in Managing Cardiovascular Disease in Selected Rural and Peri-Urban Clinics in Limpopo Province, South Africa

**DOI:** 10.3390/ijerph18052570

**Published:** 2021-03-04

**Authors:** Mamoeng Nancy Kgatla, Tebogo M. Mothiba, Tholene Sodi, Mpsanyana Makgahlela

**Affiliations:** 1 Faculty of Health Sciences, University of Limpopo, Sovenga 0727, South Africa; tebogo.mothiba@ul.ac.za; 2 Department of Psychology, University of Limpopo, Sovenga 0727, South Africa; tholene.sodi@ul.ac.za (T.S.); mpsanyana.makgahlela@ul.ac.za (M.M.)

**Keywords:** experiences, management, nurses, cardiovascular diseases, clinics

## Abstract

Deaths caused by cardiovascular diseases (CVDs) account for 60% of all deaths that occur in rural and remote areas. Disease management programs are increasingly used to improve the effectiveness of chronic care. Nurses are a key component of the health workforce and have an important role to play in CVD prevention, treatment, and the care of sick people in remote areas. Due to the nature of their work, nurses are prone to working hard, and to experience burnout, sleep, or eating disorders. This is often exacerbated by a shortage of staff and equipment. The objectives of the study were to explore and describe the experiences of professional nurses in managing CVDs in South African rural and peri-urban clinics. A qualitative, explorative-descriptive design and a contextual research approach were adopted for the present study. Purposive sampling was employed to recruit nurses who were managing patients with CVD from 11 primary health care facilities. Data were collected through semi-structured individual interviews and analyzed using Tesch’s open coding method. Interview transcripts were coded and analyzed for common themes. The following two major themes emerged from the data: perceived institutional challenges affecting the management of CVDs and nurses’ perceptions of patient challenges that impede the effective management of CVD. The study concludes by highlighting that apart from a resource challenge, the shortage of nurses in rural clinics is the biggest reason behind overcrowding, waiting long hours for consultations, and an increase in the workload, resulting in medical errors and poor quality care. It is, therefore, recommended that, for improved care and management of CVD in rural populations, local governments need to employ more skilled nurses whilst availing the necessary material resources.

## 1. Introduction and Background

According to the World Health Organization (WHO), cardiovascular diseases (CVDs) are the world’s biggest killers, causing an estimated 35 million deaths each year. This accounts for 60% of all deaths globally [1]. Up to 80% of heart disease, stroke, and type 2 diabetes mellitus (T2DM), and over one-third of cancers could be prevented by eliminating shared risk factors, mainly tobacco use, unhealthy diet, physical inactivity, and the harmful use of alcohol [2]. Premature deaths that have been caused by CVDs in people of working age (35–64 years) are expected to increase by 41% between 2000 and 2030 [3]. It has been speculated that the negative economic impact of this prediction will have a serious impact on the country. Though CVDs are viewed as diseases of the developed world, they are increasing in low and middle-income countries (LMICs). On the African continent, especially in sub-Saharan countries, CVDs are reported to account for 9.2% of the population’s total deaths. This is even more prevalent for those aged 45 years and over, which constitutes 7–10% of all adult admissions to hospitals [3]. A South African study by Rheeder, Morris-Paxton, Ewing, and Woods (2017) found that there was a high prevalence of hypertension amongst women at 50%, whilst men were at 43%, while those who were 55 years and older persons were at 50% risk [3]. In another study, Alberts, Urdal, Steyn, Stensvold, Tverdal, Nel, and Steyn (2005) revealed that in South Africa, CVDs are responsible for almost one in six deaths (17.3%) [4]. It has also been reported that there is an 8.5% prevalence of diabetes and a death rate of 215 people as a result of heart disease [5]. In South Africa, it is indicated that CVDs represents a leading threat to human health. For instance, in Limpopo Province (South Africa), a 20% prevalence for cardiovascular diseases has been reported [6]. Terzic et al. [7] study reported a high prevalence of chronic disease risk factors amongst the poor black community in the rural areas of Limpopo Province, South Africa. 

The increasing prevalence of these avoidable diseases in LMI countries has severe health and psychosocial impacts on individuals at different levels. CVDs coincide with the rise in urbanization, industrialization, and economic transition. This results in health services not always being adequately equipped to deal with the rising health issues, thus resulting in more people becoming ill and dying from CVDs. To prevent the increasing numbers of CVDs, South Africa should invest in preventative measures to curb these lifestyle diseases [8].

According to Maimela et al. [9] there are several challenges experienced by nurses and other health care workers in providing quality care for patients living with CVDs in South Africa. Some of the challenges are poor knowledge of CVDs, shortage of resources, and the long waiting times patients experience at primary health care clinics (PHC). Nurses are the main health workforce, especially in primary health care. They provide health promotion, disease prevention by educating patients, guidance on immunization, screening, dietary guidelines, medications, and ante- and postnatal care [7]. With a range of approaches to disease prevention, nurses can detect problems before they start. They can also educate patients on ways to minimize or eliminate the risk factors. The recruitment and retention of the health workforce, more especially nurses, is a critical issue facing many rural and remote areas [9]. Solving the inequitable distribution of the health workforce is an issue for both developing and developed countries [10]. The inequitable distribution may cause delays in the services rendered to patients in that area, especially older patients who may have problems with accessing the already depleting health services. This situation makes it more difficult to attain improved patient outcomes [11]. Creswell et al. [12] indicated that there was also a substantial under-treatment by general practitioners of patients with a high risk of CVDs. Rural patients get fewer prescriptions for critical medications used in the treatment of CVDs such as beta-blockers, ACE inhibitors, statins, and warfarin, despite similar or a higher burden of disease than in metropolitan areas [13].

Unless addressed with urgency, the mortality and disease burden from these health problems will continue to increase in South Africa and other countries [13]. In an attempt to solve this emerging problem of CVDs, South Africa has established primary health care services at secondary and primary health care levels that can address these serious challenges [14]. It was against this background that the study was aimed at exploring the experiences of nurses in the management of CVDs in the primary health care setting. The current study was conducted in selected rural areas of Limpopo Province.

## 2. Materials and Methods

### 2.1. Study Design

This was a qualitative study that is explorative and descriptive in design and contextual in approach. The design was deemed appropriate as it allowed professional nurses to describe their lived experiences with regard to the phenomena under investigation. By adopting a contextual approach, the authors were mindful that the study was going to be operationalized in two contexts (i.e., a rural-and-township setting), which would present with unique social, cultural, historical, and individual realities [14]. In the rural area, it required the researchers to consult and seek gatekeeper permission from the relevant tribal authorities in addition to the permission that was granted by the local government while in the peri-urban area, the researchers went ahead to access the clinics only after obtaining permission from the local government. This design was therefore deemed appropriate based on the nature of the reality being investigated, whilst allowing flexibility for it to be employed in the two geographically different study areas.

### 2.2. Study Area

The study was conducted in the Capricorn District, which is one of the five districts constituting Limpopo Province. The Capricorn District spans a surface area of about 21,705 km^2^. Seven clinics were based in Ga-Molepo, a rural area and another four in the Seshego area, a peri-urban setting, located in an area immediately adjacent to the city of Polokwane. The two study areas are about 62 km apart from each other [15]. A previous study conducted in the Ga-Dikgale (another rural area adjacent to Ga-Molepo) showed a high prevalence of CVD in people who were 45 years and above [5]. The current study, therefore, seeks to provide comparative data but with an emphasis on nurses’ experiences concerning CVD management in Limpopo’s rural and peri-urban areas. These eleven primary health care facilities were purposively selected as they are included in a bigger European Commission-funded study, known as Scaling-up Packages of Interventions for Cardiovascular disease Prevention in selected sites in Europe and Sub-Saharan Africa (SPICES).

### 2.3. Population and Sampling

The study population comprised of eighty-seven professional nurses working with CVD patients in the two selected primary health care clinics, which are situated in Limpopo Province in the Capricorn District. The study adopted the purposive homogenous sampling technique to select 19 professional nurses providing primary health care services in the selected areas. The participants included in the study were selected based on their experience with CVD management and their availability on the day fieldwork was conducted in the respective clinics. The rural clinics run on a limited staff compliment, and as a result, they have to render CVD related services on specific days of the week; ranging between 2–3 days. Only 1-to-2 professional nurses were employed in some of the clinics, causing delays in the treatment of patients. The selection criteria also targeted those nurses who had worked in the selected clinics for more than two years. The justification for the use of purposive sampling is that it enables the researcher to select participants who have insight and understanding about the phenomenon [16]. The study included all the professional nurses at the clinics who have been providing primary health care services (including chronic disease management) between two to 25 years. Sampling continued until a point of saturation, as recommended by Burns et al. [16].

### 2.4. Data Collection and Analysis

Data were collected over 20 days between March and April 2018. In-depth interviews using a semi-structured guide were conducted by the first author (also referred to as primary researcher) and an assistant researcher. The participants were requested to share their experiences of managing CVDs in their respective clinics. Probing questions were asked to enable participants to clarify issues that were unclear to the researchers. The interview guide was written in English, with a central opening question and followed by probing questions as deemed necessary. The central opening question was: “Kindly describe your experiences in providing care to patients with CVDs in the clinic”. Probes were used to enrich and elicit more responses regarding CVD management including day-to-day experiences. This process allowed for a deeper understanding of the nurses’ experiences in providing care to patients with CVDs [17]. The interviews were audio-recorded by the primary researcher and transcribed by the assistant researcher.

The study adopted Tesch’s eight steps to analyze data, provided by Creswell et al. [12]. To gain a better understanding and to derive the meaning of the whole, the primary researcher started by reading through all the transcribed data. This highlighted ideas about the data themes and how to interpret their meanings. The data collected was scaled down to codes, based on the existence or frequency of concepts used in the verbatim transcriptions. The topics that emerged during the scaling down were listed, with similar topics grouped. The topics that did not have associations were clustered separately. After going through the transcripts several times (following Tesch’s steps), themes and sub-themes were developed from the coded data and the associated texts. The data belonging to each theme were assembled in one column and a preliminary analysis was performed. This was followed by a meeting between the primary researcher and independent coder to reach a consensus on themes and sub-themes that each one had arrived at independently.

### 2.5. Measures to Ensure Trustworthiness

To ensure trustworthiness, the researchers adhered to the principles of confirmability, dependability, transferability, and credibility. This was ensured by prolonged engagement with the participants and by visiting the clinics before conducting the interviews. The appointments made enabled the research team to become familiar with the participants during the interaction and interviews, thus building a trusting relationship. Credibility and confirmability were ensured by collecting data from the nurses themselves who care for the patients with CVDs. To further strengthen the study validation, member checking was done because after the initial data collection, the primary researcher went back to the professional nurses with the transcripts to correct any errors and to add additional information. This process also assisted in assessing the overall adequacy of the data in addition to individual data points. Validation of results was also done by sending the voice recordings and the transcripts to an independent coder who was an experienced qualitative researcher. Thereafter, a meeting was held between the researcher and the independent coder and a consensus on the themes and sub-themes identified was reached. To ensure transferability, the methodological steps where adopted and a detailed description of the research method was documented.

### 2.6. Ethical Considerations

Ethical clearance was obtained from the University of Limpopo’s Turfloop Research Ethics Committee (TREC/318/2019:19). Permission to access the clinics was obtained from the Limpopo Provincial Department of Health and the Capricorn Health District Office. In the rural villages, gate-keeper permission had to be gained from local tribal authorities. Upon gaining access to clinics and participants, informed consent was obtained from all participants before each semi-structured interview session could commence. The aim and nature of the study were explained to the participants before data collection. The names of the participants were not used, but numbers were allocated to each participant to ensure anonymity and confidentiality. Anonymity was ensured by using numbers as participant identifiers. Participants were further informed that their names will be protected in the presentation and publication of the study. They were also informed that they could withdraw from the study at any time, without any repercussions.

## 3. Results

The following two themes and six sub-themes emerged during data analysis (see Table 1): (1) Perceived institutional challenges affecting management of CVDs; and (2) Nurses’ perceptions of patient challenges that hamper the effective management of CVDs.

### 3.1. Theme 1: Perceived Institutional Challenges Affecting Management of Cardiovascular Diseases (CVDs)

The findings suggest that three key institutional challenges impacted negatively on the ability of nurses to manage CVDs in the selected clinics. The identified challenges (sub-themes) are a shortage of human resources; a shortage of material resources; and, the expectation that nurses should perform the duties of other professionals. The three themes are discussed below.

#### 3.1.1. Sub-Theme 1.1: The Shortage of Human Resources Being Experienced

The findings revealed that nurses were struggling to manage CVDs or rendered substandard care because they were short-staffed. The performance of any health care system is dependent on the quality of care afforded by the nurses. Hence in this setting, a shortage of nurses was described as one of the factors resulting in substandard levels of health care. This is supported by the following participants’ excerpts:

Participant 0I: “If possible, please, let them hire more nurses especially professional nurses because patients end up having to come to the clinic early in order to avoid being consulted late and having to commute back home late after they would have been treated. They also transferred had to transfer some nurses who were vital to our clinic to Zone 4 clinic, this resulted in us being understaffed and overworked”.

The issue of being understaffed was associated with inefficiencies and politics in systems of governance at a local level. This is further supported in the following extracts by some of the nurses:

Participant 07: “There are many challenges especially the shortage of staff in this clinic, we are understaffed because we have two sisters who have left and have not been replaced. This created a big gap. It also interfered with our daily care of our patients. If they can replace them the quality of care will improve because at the moment it is not satisfactory due to this problem”.

This finding highlights that nursing staff shortages negatively impact the quality of care for people living with CVD who consult at the participating clinics.

Participant 08: “Yes, I understand that there are not enough nurses to cater for all of these patients but why the government is not hiring more nurses because sometimes we get angry if junior nurses sit down to rest or assist emergency because one day I was very angry started looking for a manager phones because I didn’t see the junior nurse and it was 10h00. Not even one patient been taken vital signs and I was busy with ante-natal care not even aware that she is busy with visitors and emergency as she was the one on the emergency room and doing triage”.

Another participant with a similar experience had this to say:

Participant 09 said: “The main problem is there is a shortage of nurses especially professional nurses because most of the time were are two on duty as professional nurses, and if one can be on sick leave only one will be left to care for all the chronic, acute patients and also pregnant women and this will compromise quality care and also cause burnout to nurses”.

#### 3.1.2. Sub-Theme 1.2: The Shortage of Material Resources Experienced

The findings further revealed that the shortage of resources negatively affected clinical outcomes and the effective management of CVDs in participating clinics. Two participants highlighted this as follows:

Participant 01: “There is a serious shortage of B.P. machines and glucometers in this facility. You will find that we share one B.P. meter in four consulting rooms. This becomes a challenge as it is very difficult for nurses to treat patients with the shortage of equipment”.

Participant 04: “We sometimes experience a shortage of medication in this clinic, and patient’s blood pressure rises due to lack of treatment. We’re just appealing to the government to send us treatment so that we can be able to control these BPs and prevent complications like CVD to our patients”.

It is therefore evident that a shortage of material resources, either due to the unavailability or non-functioning of equipment, is perceived as a barrier preventing the delivery of quality health services.

#### 3.1.3. Sub-Theme 1.3: The Expectations That Nurses Perform Duties Outside Their Scope of Practice

Another finding linked to institutional challenges was that due to diverse cases presenting at the clinic including CVD cases, nurses ended up taking on the roles of other health professionals such as medical practitioners, pharmacists, dieticians, and psychologists in the PHC clinics. What created the situation was that in some clinics, there were no visiting medical officers or allied health care workers when compared to some facilities that had at least some health professionals who would visit on some day/s of the week. Here is what some participating nurses had to say:

Participant 016: “Unfortunately, there is no visiting medical doctor in our clinic. As nurses, we are treating, issuing treatment, and perform other related work. Sometimes there is only one professional nurse at work and can’t cope and will have to refer patients to go see a doctor or dietician at Mankweng Hospital. The pharmacy assistant visits every Wednesday therefore, as nurses we have to do pharmacists work on other days”.

Participant 019: “We refer our patients who need to see the doctors to Mankweng Hospital. The pharmacy assistant visits every Wednesday, and the physiotherapist, speech and hearing therapist maybe once a month. We do not have an environmental officer and a health promotion practitioner. We liaise with the district if we seriously need them. The dietician that we had, unfortunately, has resigned. So, for now, we don’t have, but other facilities do have dieticians and visiting medical doctors. In the absence of all these professionals as nurses, we need to assist the patients and gives them service accordingly”.

Participant 03: “My wish is to add more staff in the clinic. We must have an administrative assistant because they can open patients’ file for every visiting patient to relieve nurses form doing that. This is a normal procedure that needs to happen at each clinic. If you have got only one data-capturer it is hard for nurses, it means we are also supposed to open patient files”.

It is further evidence that the shortage of key health professionals (e.g., medical officers, pharmacists, dieticians, psychologists) in the management of CVDs also contributes to the poor management of cases in rural and township primary health care facilities. This may suggest that the already overworked nursing staff, due to the lack of human and material resources, are at risk of burnout. This situation is likely to lead to the further poor management of CVD cases presenting in such facilities. These PHC facilities are the first point of contact of health care delivery with links to higher levels within the health system and other services. It was evident in this study that some of the facilities ended up having to refer their patients to a local tertiary hospital for better CVD management [18]. Unfortunately, delays in access to health care could constitute poor care for patients or delayed responses. This means that the burden of chronic diseases would continue affecting the individual patient in terms of lost productivity, cost of care as well as social aspects [18].

### 3.2. Theme 2: Nurses’ Perception of Patient Challenges That Hamper Effective Management of CVDs

During the interview sessions, the nurses referred to challenges that patients face when visiting the clinics and which in their view negatively impacted good management of CVDs. These challenges were translated into the following three subthemes:

#### 3.2.1. Sub-Theme 2.1: Patients Experience Long Hours Waiting at the Facilities

The clinics receive large numbers of patients including those with CVDs who come daily for consultation. Due to the large numbers, patients wait for long periods before they are attended to by the nurses. The long queues are also a result of routine activities such as cleaning and checking medical stock that nurses are expected to perform every morning. This observation is supported by the following excerpts:

Participant 06: “As nurses, what bothers us is that, patients come here as early as 05h00 in the clinic and sit in long queues. The cleaner starts at 08h00 because start cleaning; then patients are supposed to wait to be seen after the cleaning which ordinarily takes about 2-h. Then, as nurses, we start helping patients very late. Look, even now we have not yet started with patients’ consultation, and it is already late, look is 09h00”.

Participant 012: “I suggested that cleaners should clean after we nurses have consulted patients. Late in the afternoon so that the following day we can always start early to see patients. This is because we pity patients who come here and get their treatment late when they have arrived in the early hours of the morning. Honestly, the patients wait for long at this clinic and it is not a pleasant thing to observe daily”.

Participant 018: “My opinion is that when we come here in the morning and start working around 09h00 we should start working at 07h30 so that patients can at least receive their treatment early so that those who stay far away from the clinic can arrive early because they will not be able to manage come the following day if they return when the clinic can close prior for them being examined”.

This particular finding highlights one systemic issue standing in the patients’ way of good CVD management. The long queues and delayed time of being attended to could both contribute to poor patient self-management including adherence to CVD medications.

#### 3.2.2. Sub-Theme 2.2: The Lack of Self-Management Skills of CVD Patients

The study also found that nurses expressed a need for patients with CVDs to be guided and motivated upon diagnosis on how to manage the condition themselves. Some of the specific steps that they recommended for patients include regular visits to the clinic to check vital signs such as blood glucose, and maintaining a physical activity program, which is useful in maintaining their quality of life. The participants further emphasized the need for home-based carers to make follow-up visits with the diagnosed patients for regular screening of their BPs, diets, and glucose levels. This was considered better than just giving patients health education, which they easily forget. This is what some nurses had to say:

Participant 011: “I suggest that homebased carers visit CVD patients at home so that they can check on them and observe how they are taking their treatment; it is suspected that they are not taking them as instructed. They must also check them on the type of food they are taking because they seem not to be taking food that is good for their hearts. What I am saying is that patients with CVDs are not managing themselves well though they are taught on how to manage themselves when they come to the clinic”.

Participant 010: “Most of the patients do not follow instructions because when you ask them how they take treatment they are always not sure and also, they like to know the signs of high or low blood sugar even when health education is given daily on those conditions; so, there’s a need for them to be monitored at home by trained community health personnel to check their sugar levels and continue with health education at home”.

Participant 015: “Most of the patients are not stable because most of the time they run out of treatment. Even here we do run out of stock, and those who can’t afford to buy they relapse or complicates because they will tell you, ’I didn’t have treatment for two months either by not collecting treatment at the clinic or lack of money to buy at the chemist’. It is a problem; how will they manage themselves in those situations”.

Self-management is one of the key strategies for good CVD management and outcomes. It is evident that in the context of the present study, nurses experience poor patient self-management as another barrier to the effective management of CVDs. It is, therefore, suggested that the recommended strategy of deploying trained community health care workers to conduct home visits to monitor, deliver health education on CVDs, and checking on patients’ adherence to medication should impact patient CVD management. The timeous delivery of medications to clinics could improve the management of CVDs in these rural and township communities, thus eliminating the imminent risk linked to the shortage of medications in some clinics. This issue is beyond the nurses, community health care workers, and individual patients themselves.

#### 3.2.3. Sub-Theme 2.3: The Overcrowding at Health Facilities Leads to Poor Management of CVDs by Nurses

Participants pointed out that overcrowding at health care facilities led to poor disease outcomes, long waiting hours, and prolonged pain and suffering for patients. This was supported by the following description from participants:

Participant 008: “There are many patients collecting treatment at the clinics, and due to shortages of medications, they sometimes go home empty-handed. Sometimes we experience shortages of chairs to sit on, and you find that patient’s condition is bad but you still have to follow the queue unless if they are critical. The government must hire a lot of nurses who can be able to cater to these patients and relieve them from the frustration of overcrowding”.

Participant 011:”.You know what is bad in this clinic? Look at your watch, what time is it? Is 10h00 but because we are not many it does not look as if the queue is moving but we have been working since from the morning, people are many that are sick, and we are not managing to care for them”.

Apart from poor outcomes for patients, the participating nurses indicated that overcrowding in the clinics resulted in employee burnout, dissatisfaction, discomfort, increased medical errors, and possible reduction in patient safety. Taken together with the many other institutional management related challenges, overcrowding in health care facilities resulted in poor access to quality health services and in some cases, nosocomial infections due to poor hygiene practices [19].

## 4. Discussion

The present study sought to explore and describe the experiences of professional nurses in managing CVDs in South African rural-and-township clinics.

It was evident from the findings of the present study that there are many challenges experienced by nurses who provide care for patients with CVDs.

The findings suggest that three key institutional challenges impacted negatively on the ability of nurses to manage CVDs in the selected clinics.

A shortage of human resources was one of the challenges experienced by professional nurses in managing patients with CVDs. This affected the quality of care provided to patients with CVDs. At an institutional level, some of the key challenges to mitigate are the lack of human and material resources. The findings partly lend support to a previous study that also found that there were critical shortages of medical equipment at a rural district hospital in South Africa [19]. Unfortunately, these shortages lead to errors in CVD management, and higher mortality and morbidity rates. Unwittingly, as was supported in this study, available nurses are forced to assume multiple roles (some of which they are not trained to handle) and work extended shifts to help out in these situations. A consequence of this are nurses who are overworked and suffer burnout [20]. A similar finding was reported in one study in which it was found that over 60% of nurses admitted to hospitals were suffering from burnout. These negative experiences with CVD management put our systems of quality care at risk. Moyimane et al. [21] indicates that the shortage of nurses are impacting patient care and health care service delivery as nurses will be overworked and over-scheduled and will become stressed out and more prone to making mistakes. Nurses in these situations are likely to commit medical errors that may lead to poor quality patient care. This may result in a variety of preventable complications including medication errors, overcrowded emergency rooms, and increased mortality rates.

The findings suggest that a shortage of resources (e.g., blood pressure monitors, glucometers, and medications) puts a strain on effectively preventing and managing CVDs. This negatively affects clinical outcomes and the effective management of CVDs.

The World Health Organization has estimated that between 50 to 80 percent of medical equipment in developing countries is not functioning. The present study lends support to this observation by the World Health Organization. Most countries in the developing world lack technology assessment systems and regulatory controls to prevent the importation of inferior medical equipment. This lack of resources exposes the countries affected to dishonest market practices that put patients’ lives at risk [19]. Most countries in the developing world lack technology assessment systems and regulatory controls, which are equally important in the health care of patients. Primary health care facilities are more than just a basic set of services, they are also a key entry point for people in townships and rural communities to accessing important health care. Any obstacle in their day-to-day running (due to costs and lack of resources, poor management, and political interferences, for instance) could lead to poor quality care and loss of life. It, therefore, becomes pivotal for governments to capacitate and support township and rural-based clinics to enable them to better serve and respond to the care of CVD patients. Capacitation and support could be a strategy to help curb the growing pandemic of CVDs in developing countries. Many patients with non-communicable diseases including CVDs struggle with self-management. This results in poor disease control, reduced quality of life, and poor psychological well-being of the patients [20]. The motivation for self-management of diagnosed CVD patients is poor when compared to Tuberculosis, HIV (Human Immune Virus), and AIDS (Acquired Immune Deficiency Syndrome) management, therefore, there is a need to emphasize self-management of these patients to improve their quality of life [22]. In this study, nurses also experienced poor patient self-management to be a barrier to CVD management. Apart from medications running out of stock in the facilities, patients themselves failed to collect prescriptions at the facilities. In some cases, they failed to adhere to the proper diet, probably due to a lack of understanding of the importance of a proper diet in CVD management. To deal with challenges associated with patient self-management, task-shifting, which nurses in the present study also supported, has been recommended as a strategy to be considered [20]. In this regard, home-based carers could be empowered and encouraged to do home visits intended to improve CVD patients’ self-care and disease management whilst encouraging them to take an active role in their health care [23]. Most of the communities did not engage in self-management and prevention due to ignorance of the information given at the facilities or lack of knowledge. The involvement of home-based carers could release some of the work pressure on professional nurses working in rural and township clinics.

The findings revealed that due to many chronic diseases including CVDs, nurses are supposed to take on the roles of other health professionals such as medical practitioners, pharmacists, dieticians, and psychologists. Maier et al. [22] state that nurses performing clinical activities that have traditionally been reserved for physicians is a phenomenon that is increasing worldwide. The increase in chronic conditions has triggered adaptations to service delivery and workforce composition. Traditional occupations like nurses have expanded their scope-of-practice to fill gaps or alleviate shortages.

Although the South African Department of Health introduced the Ideal Clinic, which aimed to improve the primary health service through the reduction of long waiting hours. This was reported to be one of the challenges in the current study. Oche et al. [24] reported that the amount of time a patient waits to be seen is one factor that affects the utilization of health care services. Patients perceive long waiting times as a barrier to obtaining services. Keeping patients waiting unnecessarily can be a cause of stress for both patient and health care providers. The waiting time is a tangible aspect that patients will use to judge health personnel even more so than their knowledge and skill [22]. The waiting time is one of the parameters used in measuring the level of care received by patients [25].

## 5. Recommendations for Better Cardiovascular Disease (CVD) Management in Rural and Township Clinics

The following recommendations are made based on the study findings:

- To address the challenges that nurses are facing, facilities need to be adequately resourced to deliver high-quality service for the benefit of all their patients.
- The government needs to provide adequate human and material resources by appointing more nurses and other health professionals in these primary health care facilities.
- Continuous training for nurses should be provided to empower them to effectively care for patients with CVDs.
- The government’s procurement processes should be significantly improved to facilitate the timely receipt of ordered material resources and surgical supplies at the clinics, as expected. Additionally, the government needs to supply the clinics with the relevant material and human resources that are required to provide care to all patients including those with CVDs.
- Finally, the government should commit to eliminating nepotism and corruption in areas such as recruitment for positions and awarding of tenders for services.

## 6. Study Limitations

The present study employed a qualitative, explorative, and descriptive design with a contextual approach. This suggests that the findings of the study may not be generalized. It is therefore recommended that qualitative studies be conducted in similar settings in the future to help with the extrapolation of findings. In some clinics, more than one interview was conducted. This introduces the possibility that such an arrangement could have resulted in some form of bias since some nurses were interviewed longer than others. Caution is therefore necessary when interpreting the results for those particular settings.

## 7. Conclusions

The study concludes by calling for the government to support nurses who are working at the clinic level so that they can appropriately care for patients with CVDs. Findings of the study identified that there was a shortage of human resources like nurses, cleaners, and other members of a multidisciplinary team (e.g., a pharmacist, dietician, and visiting doctors). The provision of material resources is also a serious challenge such as basic screening equipment like BP machines and glucometers are not readily accessible for screening purposes in some of the facilities. Some participants suggested that screening equipment could be utilized by trained volunteers to empower the members of the households and to increase self-management skills. While this can be a great help, it can lead to serious challenges as there are currently shortages in the facilities. It is crucial to support and empower PHC facilities managed by professional nurses by making provision for multidisciplinary teams of health care professionals to visit the clinics, based on the needs of the facilities. The services of CHWs should be strengthened as these cadres of the workforce form a vital link between the community and the health care facility. Overcrowding and poor infrastructure also contribute to poor quality care as findings showed that due to a shortage of nurses, there is overcrowding of patients. They queue for a long time without proper infrastructure (e.g. no shelter during the rainy season and on hot summer days). This leads to patients experiencing complications due to being exposed to excessive heat or cold. Due to their workload, nurses become exhausted and suffer burnout. This leads to poor quality care, absenteeism, and dissatisfaction, which results in patients experiencing higher mortality and morbidity rates. The study concludes by calling for the government to support nurses who are working at the clinic level so that they can appropriately care for patients with CVDs.

## Figures and Tables

**Table 1:** Themes and sub-themes of nurses’ experiences during the management of cardiovascular diseases (CVDs).

Theme	Sub-Theme
1. Perceived institutional challenges affecting management of CVDs	1.1. The shortage of human resources being experienced
	1.2. The shortage of material resources being experienced
	1.3. The expectations that nurses perform duties outside their scope of practice
2. Nurses’ perceptions of patients’ challenges that hamper effective management of CVDs	2.1. Patients experience long hours waiting at facilities
	2.2. Lack of self-management skills by CVD patients
	2.3. Overcrowding at health facilities leading to poor management of CVDs by nurses

## Data Availability

The data supporting the findings of this research are available on request.
